# Perceptual Category Judgment Deficits are Related to Prefrontal Decision Making Abnormalities in Schizophrenia

**DOI:** 10.3389/fpsyt.2013.00184

**Published:** 2014-01-06

**Authors:** Thomas W. Weickert, Alejandro Terrazas, Llewellyn B. Bigelow, Jose A. Apud, Michael F. Egan, Daniel R. Weinberger

**Affiliations:** ^1^Clinical Brain Disorders Branch, NIMH/NIH, Bethesda, MD, USA; ^2^School of Psychiatry, University of New South Wales, Randwick, NSW, Australia; ^3^Neuroscience Research Australia, Randwick, NSW, Australia; ^4^Advanced R&D, MSci, Nielsen, San Francisco, CA, USA; ^5^Lieber Institute for Brain Development, Baltimore, MD, USA

**Keywords:** schizophrenia, perceptual category judgment, prefrontal cortex

## Abstract

Previous studies of perceptual category learning in patients with schizophrenia generally demonstrate impaired perceptual category learning; however, traditional cognitive studies have often failed to address the relationship of different cortical regions to perceptually based category learning and judgments in healthy participants and patients with schizophrenia. In the present study, perceptual category learning was examined in 26 patients with schizophrenia and 25 healthy participants using a dot-pattern category learning task. In the training phase, distortions of a prototypical dot pattern were presented. In the test phase, participants were shown the prototype, low and high distortions of the prototype, and random dot patterns. Participants were required to indicate whether the presented dot pattern was a member of the category of dot-patterns previously presented during the study phase. Patients with schizophrenia displayed an impaired ability to make judgments regarding marginal members of novel, perceptually based categories relative to healthy participants. Category judgment also showed opposite patterns of strong, significant correlations with behavioral measures of prefrontal cortex function in patients relative to healthy participants. These results suggest that impaired judgments regarding novel, perceptually based category membership may be due to abnormal prefrontal cortex function in patients with schizophrenia.

## Introduction

Category learning and decisions or judgments regarding ensuing category membership are cognitive processes that are integral to our daily lives. Category formation and decisions regarding category membership have been studied extensively in healthy adults. There are two principal hypotheses regarding the cognitive underpinnings of category learning. The prototype hypothesis suggests that information about category membership yields a prototype (or an average of instances), which is stored separately from the individual items contributing to formation of the prototype (1–4). Conversely, the exemplar hypothesis suggests that information regarding category membership may be an emergent factor such that information about common features may accumulate gradually and category formation may occur as a product of the number of exemplars stored in memory (5–8). Since category learning based on verbal features may be confounded by preexisting knowledge from extant categories, some research on novel category learning has focused on the formation of novel, perceptually based categories. Posner and colleagues (1, 9, 10) introduced a dot-pattern category learning procedure that provided a reductionistic approach to category formation by utilizing ill-defined dot patterns that could be grouped into categories.

Smith et al. (11) and Koenig et al. (12) have shown that distinct neural circuitry is activated whether artificial, verbal categories are derived on the basis of rules (occipital-parietal, prefrontal cortices) or similarity to recalled exemplars (occipital-parietal, temporal cortices). Results from other functional magnetic resonance imaging studies examining perceptual category learning in healthy young adults have demonstrated that a neural network encompassing bilateral prefrontal (BA 47, 8, 10), temporal (BA 21, 41), and parietal (BA 40) cortices are activated while extrastriate visual (BA 19) and posterior occipital (BA 17, 18) cortex is deactivated during the process of making judgments regarding membership in novel, perceptually based categories (13–17). The neural system associated with the learning of novel, perceptually based categories has been referred to as the Perceptual Representation System (18), because the learning that occurs is thought to be primarily dependent on occipital cortex function. Previous studies leave open the question of what are the relative contributions of prefrontal activation and occipital cortex deactivation to decisions regarding membership in novel, perceptually based categories. Since patients with schizophrenia generally display relatively preserved occipital cortex function and impaired prefrontal cortex function on the basis of cognitive testing (19), this patient group could be potentially used to determine the relative contribution of prefrontal and occipital cortices to perceptual category judgment.

Previous studies have demonstrated visual perceptual organization deficits in a portion of patients with schizophrenia, specifically those patients diagnosed with a subtype of schizophrenia referred to as disorganized (20). These visual perceptual organization deficits are associated with increased symptom severity, poor premorbid function, non-perceptual cognitive organization impairment (21) and can be influenced by top-down feedback to early visual processing centers (20). These results support other work (19) showing impaired visual-spatial perceptual abilities in approximately 25% of a large sample of patients chronically affected with schizophrenia who display low premorbid IQ estimates. Although a minority of patients display visual-spatial perceptual abnormalities, many studies report perceptually based category judgment deficits in patients with schizophrenia, suggesting that these category judgment deficits may not be solely due to visual-spatial perceptual impairment.

Learning and judgment of category membership, both semantic and perceptual, is relevant to schizophrenia to the extent that patients with schizophrenia often display disorganized thought processing (22) and they generally display impaired cognitive processing that has been related to poor functional outcome (23). Intact thought processing is integral to category learning and decision making which would ultimately impact ability to function on a daily basis. While semantic category deficits are well documented in patients with schizophrenia, perceptual category learning and judgment has been less studied.

Studies using geometric shapes to define membership in perceptually based categories (24, 25) have shown that perceptual category learning is significantly impaired in patients with schizophrenia. In more recent findings, Keri et al. (26) showed equivalent perceptual dot-pattern category learning between patients with schizophrenia and healthy adults; however, Keri (27) also showed that patients with schizophrenia who had impaired working memory performed at chance levels with respect to decisions pertaining to perceptual dot-pattern category membership (suggestive of prefrontal contributions to judgment of perceptual categories). Since the majority of patients with schizophrenia display prefrontal impairment and relative occipital cortex preservation on the basis of cognitive assessment (19), and abilities from other cognitive domains (such as visual-spatial abilities) were not assessed in previous studies of perceptual category judgment in patients with schizophrenia, in the present study perceptual category learning and judgment were assessed in sample of patients with schizophrenia who did not display deficits on cognitive measures of visual-spatial ability (representative, in part, of occipital cortex function).

Perceptual category learning was examined in patients with schizophrenia using a version of the dot-pattern category learning task described previously (28, 29). In this task, distortions of a prototypical dot pattern are presented in a training phase during which participants are given a low-level decision task designed to ensure processing of the dot patterns (i.e., they are told to find and point to the dot that is approximately in the center of the pattern) and they are not initially informed of the true task objective. During the test phase, participants are required to indicate whether the dot-pattern presented (either prototype, low distortions, or high distortions of the prototype, or random dot pattern) is a member of the category of dot-patterns previously presented during the study phase. Based on the majority of previous studies showing impaired perceptual category judgment in patients with schizophrenia (24, 25, 27), the working hypothesis in the current study was that relative to healthy participants, patients with schizophrenia would display deficits when making decisions regarding category membership within a newly learned, perceptually based category. Additionally, as Ashby and O’Brien (18) indicate, there are no traditional cognitive data that address the role of regional cortical contributions to perceptual category judgment. Therefore, structural equation modeling was also used to test the equality of correlation matrices between perceptual category judgment and measures from other cognitive domains in patients and healthy participants. Thus, an additional novel hypothesis was that the correlational matrices pertaining to perceptual category judgment and other cognitive domains would not be equivalent between people with schizophrenia and healthy participants. Comparing the relationship among the ability to make judgments pertaining to novel perceptually based category membership and performance on tests reflecting other cognitive domains in patients with schizophrenia relative to healthy participants may provide further insight into the relative contribution of cortical regions to perceptually based category judgment.

## Materials and Methods

### Participants

Twenty-six patients, 23 males and 3 females (81% right handed) with a diagnosis of schizophrenia (21 inpatients and 5 outpatients) participated in this study. Two board-certified psychiatrists concurred on diagnosis by Structured Clinical Interview for the Diagnostic and Statistical Manual-fourth edition without knowledge of cognitive abilities. Patients that received concurrent axis I psychiatric diagnoses other than schizophrenia, or having a history of current substance abuse, head injuries with concomitant loss of consciousness, seizures, central nervous system infection, diabetes, or hypertension were excluded. Patients were classified into undifferentiated 44%, paranoid 24%, disorganized 8%, and residual 8% subtypes, as well as receiving classifications of chronic schizophrenia 4%, and schizoaffective depressed disorder 12%. The majority of patients (88%) were receiving doses of atypical antipsychotic medication such as olanzapine and risperidone at the time of testing and 8% were not receiving antipsychotic medications. In addition to patients with schizophrenia, 25 healthy participants, 12 males and 13 females (100% right handed) recruited through the National Institutes of Health normal volunteer office, participated in this study. Healthy participants with a history of psychiatric disorders, current substance abuse, head injuries with concomitant loss of consciousness, seizures, central nervous system infection, diabetes, or hypertension were excluded. All participants provided informed written consent prior to participation in this study. The Institutional Review Board of the National Institute of Mental Health provided approval for this study.

A four subtest version of the Wechsler Adult Intelligence Scale-Revised (WAIS-R) (30), consisting of the Arithmetic, Digit Symbol Substitution Test (DSST), Picture Completion, and Similarities subtests, was administered to obtain an estimate of current Full Scale Intelligence Quotient (FSIQ) (19, 31, 32). Additionally, the Reading subtest of the Wide Range Achievement Test-Revised (WRAT-R) (33) was administered to obtain an estimate of premorbid intellectual levels in patients. The Reading subtest of the WRAT-R is thought to reflect “preserved” abilities since it is a test of decoding skills which are routinely acquired prior to the onset of disease and appear to remain unaffected by the disease process in analogous fashion to the “hold” subtests (those tests that are insensitive to deterioration associated with normal aging and certain types of brain damage) of the WAIS-R (19, 34). Previous studies have consistently demonstrated reading scores to be viable measures of premorbid intellect (34–38). The Benton Line Orientation test (39) was used to determine differences between patients and healthy adults with respect to visual-spatial perceptual abilities. See Table 1 for the mean age, estimated current IQ, reading standard scores, and Benton Line Orientation scores of patients and healthy participants. Separate independent *t*-tests revealed no significant difference between patients and healthy participants with respect to Reading standard scores, *t*(38) = 0.74, *p* = 0.46, visual-spatial abilities (Benton Line Orientation test), *t*(37) = 0.52, *p* = 0.61, and a trend toward a significant difference between groups with respect to age, *t*(48) = 1.91, *p* = 0.06. As expected, there were significant differences between the groups on the basis of education, *t*(49) = 2.72, *p* = 0.01, and current WAIS-R estimated FSIQ, *t*(39) = 5.20, *p* < 0.001.

**Table 1:** **Age, education, IQ, reading scores, and symptom ratings in patients with schizophrenia and healthy participants**.

	Patients with schizophrenia	Healthy participants
Age (years)	32.2 (9.2)	36.9 (8.1)
Education (years)	13.6* (2.3)	15.5 (2.6)
WRAT-R reading	98.8 (15.4)	102.1 (8.4)
WAIS-R FSIQ	89.0** (9.9)	105.5 (9.5)
Benton Line Orientation	24.8 (6.8)	25.9 (4.4)
PANSS
Positive	13.7 (4.6)	–
Negative	15.6 (6.0)	–
General	28.1 (7.7)	–
Total	57.3 (15.2)	–

*Denotes patients with schizophrenia display significant difference from healthy participants at *p* < 0.01.

**Denotes patients with schizophrenia display significant difference from healthy participants at *p* < 0.001.

*Standard deviation is provided in parentheses. WRAT-R Reading, Wide Range Achievement Test-Revised Reading Standard Score; WAIS-R FSIQ, Wechsler Adult Intelligence Scale-Revised Full Scale IQ; PANSS, Positive and Negative Syndrome Scale Scores. Patients with schizophrenia *N* = 26, healthy participants *N* = 25*.

### Dot-pattern category learning test

**Figure 1:**
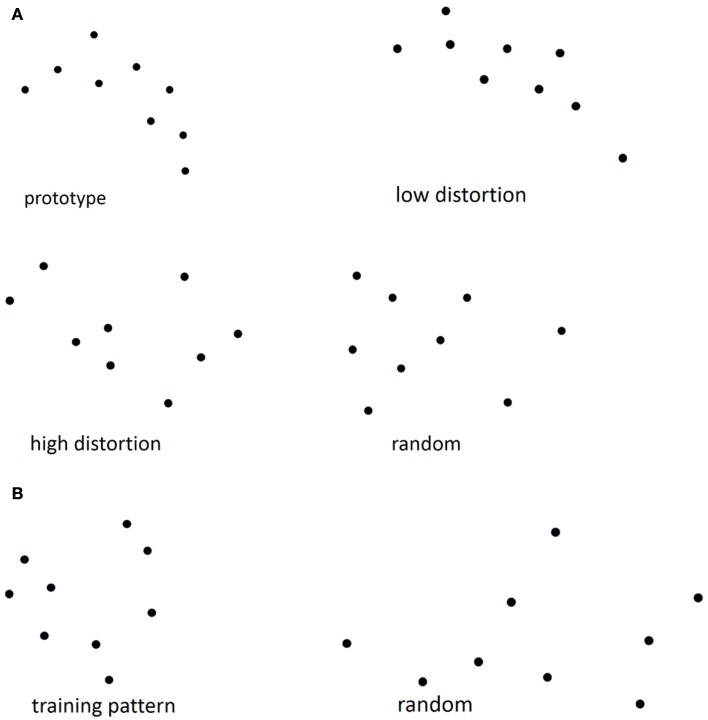
**(A)** Examples of prototype, high, low distortion, and random dot patterns from the dot-pattern judgment test, **(B)** examples of training and random patterns from the dot-pattern recognition test.

The dot-pattern category learning test used in the current study followed the specifications described previously (28, 29). The test was divided into two phases: a training phase and a test phase. First, a prototype dot pattern was constructed by randomly placing nine dots on the computer screen. New “high distortions” of the prototype were constructed by displacing each dot in a random direction at a probabilistically determined distance. All participants were administered the training phase of the dot-pattern category learning task during which they were presented with 40 new high distortions of a prototype dot pattern for 5 s each (see Figure 1A for an example). Participants were instructed to “point to the dot closest to the center of the pattern.” Five minutes after completion of the training phase all participants were told that the patterns they had previously examined “all belonged to a single category of patterns, in the same sense that, if a series of different dogs had been presented, they would all belong to the category: ‘dog’.” Participants were then randomly presented with 84 new dot-patterns consisting of 4 presentations of the prototype, 20 new high distortions of the prototype, 20 low distortions of the prototype, and 40 random dot patterns (high distortions of new prototypes) with a maximum of three consecutive occurrences of patterns from the same distortion type. Participants were instructed to make a “yes/no” judgment as to whether or not the new dot-patterns belonged to the previously examined category.

### Dot-pattern recognition test

The dot-pattern recognition test used in the present study also followed the specifications described previously (28, 29). This test was also divided into training and test phases. Approximately 1 week after the dot-pattern category learning task, all participants were administered the training phase of the dot-pattern recognition task, during which they were presented with 40 repetitions of the same dot pattern for 5 s each (see Figure 1B for an example). Participants were again instructed to “point to the dot closest to the center of the pattern.” Five minutes after completion of the training phase all participants were presented with 84 new dot-patterns consisting of 8 presentations of the training pattern and 76 random dot patterns. Participants were instructed to make a “yes/no” judgment as to whether or not each dot pattern was presented during the dot-pattern recognition training phase. The recognition task used in the present study was based on “over-learned” material so that the recognition results might be used as a “control” condition.

### Assessment of abilities from different cognitive domains

To determine relationships among dot-pattern category judgments and other cognitive abilities known to be dependent on regionally different brain systems, standard neuropsychological tests assessing abilities from different cognitive domains were administered to all patients and a subset of healthy participants over a period of one to three sessions by a psychologist or psychometrician trained in administration and scoring of all tests. Scoring followed standardized procedures. The Wisconsin Card Sorting Test (WCST) (40), a test of planning and set shifting, was administered as a test of prefrontal cortex executive function. The N-back (41) and California Verbal Learning Test (CVLT) (42) were administered as tests of prefrontal cortex working memory. The vigilance portion of the Continuous Performance Test (CPT) (43) and form A of the Trail Making Test (44) were administered as tests of attention. The paired-associates test from the Wechsler Memory Scale-Revised (WMS-R) (45) was administered as a test of hippocampal function and episodic memory. The semantic cluster ratio from the CVLT and letter (F-A-S) and category (animals-fruits-vegetables) fluency (46) were used as measures of language abilities. The Benton Line Orientation test (39) was administered as a test of occipital-parietal visual-spatial abilities. For means and standard deviations of the additional cognitive tests that were administered to patients with schizophrenia and healthy participants see Table 2.

**Table 2:** **Means and standard deviations (in parentheses) for additional cognitive tests in patients with schizophrenia and healthy participants**.

	Patients with schizophrenia	Healthy participants	*T*	*p*
General intellectual ability				
WAIS-R estimated full scale IQ	89.0 (9.9)	105.5 (9.5)	5.20	<0.001*
Prefrontal cortex/executive function				
WCST categories	3.8 (3.2)	6.5 (3.4)	2.55	0.015*
Prefrontal cortex/working memory				
N-back two back number correct	45.7 (23.7)	68.5 (13.3)	2.85	0.008*
CVLT list A 1–5 total correct	45.6 (17.3)	59.9 (6.4)	2.80	0.010*
Attention				
CPT vigilance correct	28.6 (1.7)	28.5 (3.4)	0.10	0.924
CPT distractibility correct	22.9 (6.4)	26.6 (7.4)	1.62	0.11
Trail making form A	44.4 (20.9)	30.7 (15.5)	2.15	0.038*
Hippocampus/memory				
WMS-R verbal paired-associates I total	15.7 (5.4)	18.5 (4.0)	1.71	0.096
Language				
CVLT semantic cluster ratio	1.9 (1.1)	2.1 (1.1)	0.46	0.651
Fluency F-A-S	33.2 (11.8)	45.1 (10.3)	3.09	0.004*
Fluency animals-fruits-vegetables	33.9 (11.6)	49.8 (16.6)	3.50	0.001*
Occipital-parietal/visual-spatial				
Benton Line Orientation	24.8 (6.8)	25.9 (4.4)	0.52	0.609

*Denotes patients with schizophrenia were significantly different from healthy participants.

*WAIS-R, Wechsler Adult Intelligence Scale-Revised; WRAT-R, Wide Range Achievement Test-Revised; WCST, Wisconsin Card Sort Test; CPT, Continuous Performance Test; WMS-R, Wechsler Memory Scale-Revised; CVLT, California Verbal Learning Test*.

### Assessment of psychotic symptoms

Psychotic symptom severity was assessed weekly in patients with schizophrenia using the Positive and Negative Syndrome Scale (PANSS) (47), by members of the nursing staff trained in the administration and scoring of the PANSS. PANSS assessments within 1 week from the dot-pattern category learning task testing date were used to obtain indices of positive and negative symptoms, general, and total symptom scores. See Table 1 for the mean PANSS scores for patients with schizophrenia.

### Data analyses

The mean number of correct responses made by patients with schizophrenia and healthy participants during dot-pattern category judgments of the prototype, each level of distortion, and random dot patterns were analyzed using a series of independent *t*-tests since the potential number of correct responses in each condition differed due to experimental design. An identical series of analyses were also applied to the dot-pattern category judgment reaction time data. The mean number of correct responses during dot-pattern recognition was analyzed using independent *t*-tests since there were only two variables of interest (training pattern and random dot patterns) and the potential number of correct responses in each condition differed due to experimental design. The mean reaction time of correct responding by patients with schizophrenia and healthy participants during dot-pattern recognition for the training pattern and random dot patterns were also analyzed using a series of independent *t*-tests. Results of all independent *t*-tests referred to above were determined to be significant only after application of a Bonferroni correction for multiple comparisons with α set at 0.05.

Correlations of dot-pattern category judgment correct responses with other cognitive variables and PANSS scores were performed to examine the relationship of dot-pattern category judgment scores to other cognitive abilities (primarily but not exclusively representative of different brain regions) and symptom severity. Using a standard test for correlations, correlations among variables representing different non-exclusive regions of cortical function and perceptual category judgment scores were tested to determine differences in correlation strength between groups. A factor analysis (Varimax normalized, extraction method: principal components) was used to highlight the largest correlations and denote which factors contribute significant additional variance (loadings >0.700 were considered significant). To further analyze the relationship between prefrontal cortex function and perceptual category judgments in patients and controls, the equality of correlation matrices consisting of variables representing prefrontal cortex and perceptual category judgment measures were tested with correlational pattern hypothesis testing using structural equation modeling. Correlation matrices for each group consisting of the following five variables: prototype classification correct, low distortion classification correct, high distortion classification correct, random classification correct, and WCST categories attained, were chosen to minimize the variable-to-subject ratio. In performing the structural equation modeling analysis a Generalized Least Squares (GLSs) followed by a Maximum Likelihood (ML) discrepancy function was used to perform correlational pattern hypothesis tests between samples.

## Results

### Dot-pattern category learning

**Figure 2:**
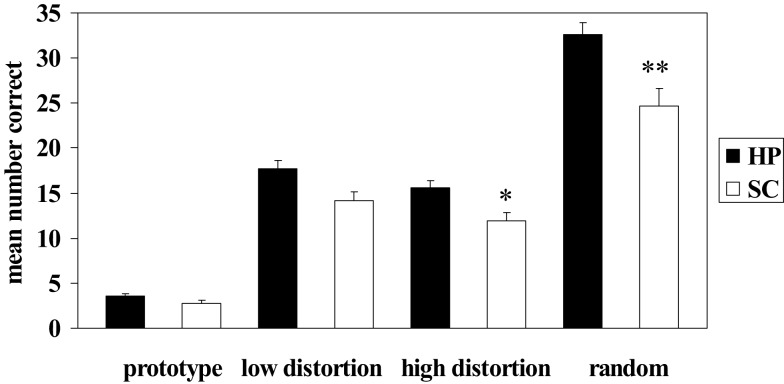
**Mean number correct during dot-pattern category judgments in patients with schizophrenia (SC) and healthy participants (HP)**. Error bars denote standard error. *SC high distortion correct significantly different from HP high distortion correct, *p* = 0.003. **SC random correct significantly different from HP random correct, *p* < 0.002.

Patients with schizophrenia displayed an impaired ability to make judgments regarding membership in novel perceptually based categories relative to healthy participants. Results from the independent *t*-tests on the mean number of correct responses during category judgments revealed significant differences between patients and healthy participants at all levels of dot-pattern distortion (see Table 3; Figure 2); however, only high distortion and random dot-patterns remained significant after application of the Bonferroni correction for multiple comparisons. Thus, patients with schizophrenia were significantly impaired relative to healthy participants with respect to their ability to make judgments regarding category membership of high distortion and random dot patterns. Results from the independent *t*-tests on the mean reaction time to correct responses during category judgments revealed no significant differences between groups after application of the Bonferroni correction (see Table 3).

**Table 3:** **Mean dot-pattern category classification and recognition learning scores and standard deviations (in parentheses) for patients with schizophrenia and healthy participants**.

	Patients with schizophrenia	Healthy participants	*T*	*p*
Dot-pattern category classification
Prototype number correct	2.8 (1.3)	3.6 (1.0)	2.33	0.024
Low distortion number correct	14.2 (5.3)	17.7 (2.9)	2.93	0.005
High distortion number correct	11.9 (4.8)	15.6 (3.9)	3.07	0.003*
Random number correct	24.7 (10.2)	32.6 (6.4)	3.33	0.002*
Prototype reaction time	2555.4 (900.4)	2356.4 (651.8)	0.90	0.372
Low distortion reaction time	2776.6 (810.1)	2253.3 (503.1)	2.76	0.008
High distortion reaction time	2870.0 (611.4)	2602.3 (525.4)	1.67	0.101
Random reaction time	2911.7 (618.1)	2725.4 (536.7)	1.15	0.257
Dot-pattern category recognition
Training pattern number correct	4.8 (1.9)	4.3 (1.6)	1.00	0.32
Random number correct	35.0 (18.0)	33.2 (5.2)	0.48	0.63
Training pattern reaction time	2719.1 (846.0)	2817.4 (567.1)	0.48	0.632
Random reaction time	2778.7 (550.2)	2598.0 (456.8)	1.26	0.213

*Denotes patients with schizophrenia were significantly different from healthy participants following Bonferroni correction with α set at *p* = 0.05.

### Dot-pattern recognition

Results from the independent *t*-test analyses of correct responding during dot-pattern recognition revealed no significant differences between patients and healthy participants during responding to the training pattern or to random dot patterns (see Table 3). Results from the independent *t*-test analyses on the mean reaction time of correct responses during dot-pattern recognition revealed no significant differences between patients and healthy participants during responding to the training pattern or to random dot patterns (see Table 3).

### Correlations among category judgment, other cognitive variables, and symptoms

In patients with schizophrenia, moderately strong, significant correlations were obtained between correct responding during dot-pattern category judgment and measures of the WAIS-R, WCST, WMS-R, CVLT semantic cluster ratio, letter fluency, and PANSS. In healthy participants, moderately strong, significant correlations were obtained between correct responding during dot-pattern category judgment and measures of the N-back, CVLT, Trail Making Test, WMS-R, category fluency, and Benton Line Orientation. See Table 4 for the correlations among dot-pattern category judgment measures and measures from other cognitive domains in patients and healthy participants. Patients with schizophrenia showed moderately strong, significant *positive* correlations among dot-pattern category judgment variables and a putative prefrontal executive function measure and moderately strong, significant *inverse* correlations among the dot-pattern category judgment random variable and language measures.

**Table 4:** **Correlations of category learning correct responding with cognitive and symptom scores**.

	Prototype		Low distortion		High distortion		Random	
	SC	HP	SC	HP	SC	HP	SC	HP
General intellectual ability
WAIS-R estimated full scale IQ	0.22	0.04	0.28	−0.07	0.40*	0.07	−0.20	0.26
Prefrontal cortex/executive function
WCST categories	0.47*	0.17	0.47*	0.18	0.64**	0.03	0.05	0.33
Prefrontal cortex/working memory
N-back two back number correct	0.09	a	0.18	0.60*	0.25	0.73*	0.13	0.67*
CVLT list A 1–5 total correct	0.11	−0.42	0.02	−0.05	0.02	−0.56*	−0.22	−0.53*
Attention
CPT vigilance correct	0.29	−0.14	0.31	−0.09	0.15	−0.02	0.11	0.30
Trail making form A	0.11	0.26	0.01	−0.13	−0.14	−0.14	0.14	−0.52*
Hippocampus/memory
WMS-R verbal paired-associates I total	0.36	−0.01	0.44*	0.38	0.41*	0.30	0.01	0.77*
Language
CVLT semantic cluster ratio	−0.12	−0.46	0.03	0.06	0.38	−0.35	−0.60*	−0.62*
Fluency F-A-S	−0.18	0.49	−0.18	−0.28	0.07	−0.11	−0.46*	0.15
Fluency animals-fruits-vegetables	−0.20	0.62*	−0.23	−0.30	0.06	−0.23	−0.13	0.03
Occipital-parietal/visual-spatial
Benton Line Orientation	−0.06	0.60*	0.06	−0.13	0.11	0.14	0.07	0.19
Symptoms
PANSS positive symptoms	−0.41*	–	−0.32	–	−0.07	–	−0.42*	–
PANSS negative symptoms	−0.32	–	−0.27	–	−0.15	–	−0.33	–

*Significant correlation at *p* < 0.05, **significant correlation at *p* < 0.01, a, no correlation calculated due to restricted range; SC, patients with schizophrenia; HP, healthy participants; WAIS-R, Wechsler Adult Intelligence Scale-Revised; WRAT-R, Wide Range Achievement Test-Revised; WCST, Wisconsin Card Sort Test; CPT, Continuous Performance Test; WMS-R, Wechsler Memory Scale-Revised; CVLT, California Verbal Learning Test; PANSS, Positive and Negative Syndrome Scale.

Between groups comparisons of the correlations among prefrontal cortex measures and category judgment variables showed a significant difference between the correlation of prototype correct with WCST categories in patients and prototype correct with CVLT correct in healthy participants, *p* < 0.001. Similarly, a significant difference was also obtained between the correlation of high distortion correct with WCST categories in patients and high distortion correct with CVLT correct in healthy participants, *p* < 0.0001. Other between group comparisons among correlations between perceptual category judgment variables and different prefrontal cortex measures were not significant.

Results of the factor analysis revealed differences between the groups with respect to factor loadings. In both patients and healthy participants, a two factor solution accounted for 55.7 and 56.1% respectively, of the cumulative variance. In patients with schizophrenia, F-A-S fluency, category fluency, CVLT 1–5 total correct, and CVLT semantic cluster ratio produced significant loadings on factor 1 (representing 32.8% of the total variance and a language/memory factor), while WCST categories, dot-pattern category prototype, low distortion, and high distortion number correct produced significant loadings on factor 2 (representing 22.9% of the total variance and an executive function/prefrontal/category decision making factor). In the healthy participants, WMS-R verbal paired-associates I total correct, 2 back number correct, and dot-pattern category random number correct produced significant loadings on factor 1 (representing 33.6% of the total variance and a frontal-hippocampal, working memory/category decision making/memory factor), while F-A-S fluency and CVLT semantic cluster ratio produced significant loadings on factor 2 (representing 22.5% of the total variance and a language factor). Results of the correlational pattern hypothesis testing using structural equation modeling of the correlation matrices suggests abnormal prefrontal cortex function during perceptual category judgment in patients with schizophrenia. There was a lack of equality among the correlation matrices of patients and controls, GLS to ML χ^2^ = 28.29, *df*  = 10, *p* = 0.002.

There was no clear differential pattern of correlations among category judgment scores and attention and visual-spatial measures between patients and healthy participants. Judgments of dot-pattern category learning also displayed moderately strong, significant correlations with positive symptoms in the patients.

## Discussion

Patients with schizophrenia displayed an impaired ability to make judgments regarding membership in novel, perceptually based categories relative to healthy participants. These results support previous work demonstrating that patients with schizophrenia display impaired judgment regarding novel, perceptually based category membership (24, 25, 27). Keri (27) used the mean categorization performance across all distortion levels to show impaired perceptual dot-pattern categorization judgment in patients with schizophrenia who had working memory impairment but they did not control for visual-spatial perceptual deficits. Thus, based on the Keri (27) finding it was not clear if there was a differential impairment related to prefrontal function with respect to perceptual category learning or if the deficit could have been explained by impaired perceptual ability. Results from the present study further suggest that the perceptual and recognition processes associated with the task can remain intact in some patients while categorization judgment processes may be negatively influenced by the disease process or its treatment. The finding of no significant difference between patients with schizophrenia and healthy participants with respect to the number of prototype dot-patterns correct, which were not previously presented and must be inferred, would suggest that the synthesis of novel perceptually based categories is preserved. Significant differences between patients with schizophrenia and healthy participants for correct responding to high distortions of the prototype and random patterns would suggest that the judgment of more difficult items from new perceptual categories is impaired in patients. Evidence from previous studies showing that patients with schizophrenia can make decisions regarding category membership for extant semantic categories in a manner that is equivalent to healthy adults (48), would further suggest, in conjunction with findings from the present study, that only the judgment of marginal members of novel, perceptually based categories is impaired in patients with schizophrenia. The lack of significant differences between patients and healthy participants with respect to mean reaction time of correct responses during dot-pattern category judgment suggests that any differential performance with respect to correct responses was not due to a generalized slowness in responding.

Healthy participants appear to rely on different cognitive processes putatively representing different lateralized regions of the prefrontal cortex during spatial versus verbal working memory (49–52) in which judgments regarding novel perceptually based categories are positively correlated with spatial working memory ability but inversely correlated with verbal working memory ability. People with schizophrenia appear to show positive correlations between judgment of novel perceptual categories and executive function ability. A recent study has shown positive relationship between the number of WCST categories attained and fractional anisotropy of the right middle frontal gyrus-striatum tract in patients with schizophrenia (53) which supports the positive relationship between WCST categories and judgment of novel, perceptually based categories in our study. Thus, results from these correlational findings from the present study suggest that patients with schizophrenia exhibit abnormal prefrontal cortex function during judgments regarding marginal members of novel, perceptually based categories. These findings were further supported using a factor analytical approach and correlational pattern hypothesis testing with a structural equation modeling analysis.

There are limitations to this study. Although these findings are suggestive of abnormal prefrontal cortical function contributing to impaired novel category judgments in patients with schizophrenia, it can not be conclusively determined which regions of the neural network associated with dot-pattern category learning and judgment are impaired in patients with schizophrenia due, in part, to the multifactorial nature of the cognitive tests used for correlational analyses. Patients with lesions in regions other than the prefrontal cortex have shown impairment on the WCST (54) and so-called medial temporal lobe patients have displayed CVLT deficits (55). Clearly, future functional neuroimaging studies of dot-pattern category learning in patients with schizophrenia and other illnesses will be necessary to delineate the neural circuitry associated with perceptually based category learning and judgment deficits. However, the correlations among category learning and the cognitive measures remain a reasonable basis for the formulation of new hypotheses regarding the nature of cortical contributions to novel, perceptually based category judgments in patients with schizophrenia.

In relation to the specificity of the deficit to visual category judgment versus an impairment that is related to increasing task difficulty, in the present study, the patients with schizophrenia displayed performances that were not significantly different from healthy participants on the vigilance and distractibility versions of the CPT and the Benton Line Orientation test, each of which represents increasing difficulty level in relation to attention and visual-spatial abilities, respectively. Thus, in this sample of patients with schizophrenia, task difficulty alone may not have been responsible for impaired perceptual category judgment.

Additionally, the heterogeneity of cognitive deficits displayed by patients with schizophrenia, often exhibiting wide-spread abnormalities across all cognitive domains tested (19), can make it difficult to isolate a specific deficit or a specific brain region associated with a particular cognitive deficit. However, that same heterogeneity can be used to advantage to assess specific cognitive deficits and associated neural regions when patient samples are selected for the lack of specific deficits. In the present study, the sample of patients displayed no significant differences from the healthy participants on measures of visual perceptual abilities (line orientation assessing visual cortex function in part), a test of recognition memory (dot-pattern recognition test assessing medial temporal lobe function in part), and tests of attention (CPT assessing anterior cingulate function in part). By selecting samples of patients with relatively restricted impairment, the ability to make assessments with respect to regional functionality may be increased.

Another limitation of the present study pertains to the effects of antipsychotic medication upon the ability to make judgments about category membership. The majority of patients were receiving antipsychotic medication prior to and during dot-pattern category learning and judgment, which may have influenced the patient’s ability to make judgments regarding category membership. However, previous studies typically show generalized improvement in cognitive abilities following the administration of antipsychotic medication (56–58). Nevertheless, future studies should also examine the ability to form novel, perceptually based categories and the ability to make judgments regarding category membership in first episode psychosis and people at high risk.

Regarding the “normal” recognition observed in patients with schizophrenia in the present study, the purpose of the recognition control task was not to suggest that patients with schizophrenia display normal recognition memory in general, but rather to show that patients with schizophrenia can display normal recognition memory for “over-learned” perceptual patterns. Results of previous recognition studies in patients with schizophrenia have produced varied results: some studies demonstrate normal recognition (59–63), other studies show impaired recognition (64–66), and some studies report both normal and impaired recognition (67–71) that appear to depend on experimental conditions, variability within the illness, and medication status. In the present study, patients were capable of recognizing the over-learned dot patterns and yet failed to make correct category judgments in relation to dot-pattern membership. No significant difference between patients and healthy participants on a measure of visual-spatial perceptual abilities also suggests that differences in perceptual abilities did not influence perceptual categorization judgment differences in the present study.

In summary, patients with schizophrenia displayed an impaired ability to make judgments regarding marginal members of novel, perceptually based categories relative to healthy participants. These findings, in conjunction with correlations among category judgment performance and cognitive measures of prefrontal cortex function in patients with schizophrenia, suggest that impaired judgments regarding novel, perceptually based category membership may be due to abnormal prefrontal cortical function in patients with schizophrenia.

## Author Contributions

Thomas W. Weickert conceived of the project, assessed all participants on the experimental task, ran the statistical analyses, and wrote the manuscript. Alejandro Terrazas supervised computer programing of the experimental and control tasks and contributed to editing the manuscript. Llewellyn B. Bigelow participated in SCID interviews and contributed to editing the manuscript. Jose A. Apud participated in SCID interviews, managed the inpatient unit, and contributed to editing the manuscript. Michael F. Egan participated in SCID interviews, managed the inpatient unit, and contributed to editing the manuscript. Daniel R. Weinberger supervised inpatient unit, the project, and contributed to editing the manuscript.

## Conflict of Interest Statement

The authors declare that the research was conducted in the absence of any commercial or financial relationships that could be construed as a potential conflict of interest.

## References

[1] Posner MI Keele SW On the genesis of abstract ideas. J Exp Psychol (1968) 77(3):353–63 10.1037/h0025953 5665566

[2] Reed SK Pattern recognition and categorization. Cognit Psychol (1972) 3(3):382–407 10.1016/0010-0285(72)90014-X

[3] Hayes-Roth B Hayes-Roth F Concept learning and the recognition and classification of exemplars. J Verb Learn Verb Behav (1977) 16(3):321–38 10.1016/S0022-5371(77)80054-6

[4] Fried LS Holyoak KJ Induction of category distributions: a framework for classification learning. J Exp Psychol Learn Mem Cogn (1984) 10(2):234–57 10.1037/0278-7393.10.2.234 6242740

[5] Medin DL Schaffer MM Context theory of classification learning. Psychol Rev (1978) 85(3):207–38 10.1037/0033-295X.85.3.207

[6] Nosofsky RM Choice, similarity, and the context theory of classification. J Exp Psychol Learn Mem Cogn (1984) 10(1):104–14 10.1037/0278-7393.10.1.104 6242730

[7] McClelland JL Rumelhart DE Distributed memory and the representation of general and specific information. J Exp Psychol Gen (1985) 114(2):159–97 10.1037/0096-3445.114.2.159 3159828

[8] Hintzman DL “Schema abstraction” in a multiple-trace memory model. Psychol Rev (1986) 93(4):411–28 10.1037/a0027023 22686158

[9] Posner MI Information reduction in the analysis of sequential tasks. Psychol Rev (1964) 71:491–504 10.1037/h0041120 14216895

[10] Posner MI Goldsmith R Welton KE Perceived distance and the classification of distorted patterns. J Exp Psychol (1967) 73:28–38 10.1037/h0024135 6047706

[11] Smith EE Patalano AL Jonides J Alternative strategies of categorization. Cognition (1998) 65(2–3):167–96 10.1016/S0010-0277(97)00043-7 9557382

[12] Koenig P Smith EE Glosser G DeVita C Moore P McMillan C The neural basis for novel semantic categorization. Neuroimage (2005) 24(2):369–83 10.1016/j.neuroimage.2004.08.045 15627580

[13] Aizenstein HJ MacDonald AW Stenger VA Nebes RD Larson JK Ursu S Complementary category learning systems identified using event-related functional MRI. J Cogn Neurosci (2000) 12(6):977–87 10.1162/08989290051137512 11177418

[14] Little DM Thulborn KR Correlations of cortical activation and behavior during the application of newly learned categories. Cogn Brain Res (2005) 25(1):33–47 10.1016/j.cogbrainres.2005.04.015 15936179

[15] Reber PJ Stark CE Squire LR Cortical areas supporting category learning identified using functional MRI. Proc Natl Acad Sci U S A (1998) 95(2):747–50 10.1073/pnas.95.2.747 9435264 PMC18492

[16] Reber PJ Stark CE Squire LR Contrasting cortical activity associated with category memory and recognition memory. Learn Mem (1998) 5(6):420–8 10489259 PMC311251

[17] Reber PJ Wong EC Buxton RB Comparing the brain areas supporting nondeclarative categorization and recognition memory. Brain Res Cogn Brain Res (2002) 14(2):245–57 10.1016/S0926-6410(02)00122-2 12067697

[18] Ashby FG O’Brien JB Category learning and multiple memory systems. Trends Cogn Sci (2005) 9(2):83–9 10.1016/j.tics.2004.12.003 15668101

[19] Weickert TW Goldberg TE Gold JM Bigelow LB Egan MF Weinberger DR Cognitive impairments in patients with schizophrenia displaying preserved and compromised intellect. Arch Gen Psychiatry (2000) 57:907–13 10.1001/archpsyc.57.9.907 10986554

[20] Uhlhaas PJ Silverstein SM Perceptual organization in schizophrenia spectrum disorders: empirical research and theoretical implications. Psychol Bull (2005) 131(4):618–32 10.1037/0033-2909.131.4.618 16060805

[21] Uhlhaas PJ Phillips WA Silverstein SM The course and clinical correlates of dysfunctions in visual perceptual organization in schizophrenia during the remission of psychotic symptoms. Schizophr Res (2005) 75(2–3):183–92 10.1016/j.schres.2004.11.005 15885509

[22] Andreasen N Scale for the assessment of thought, language, and communication (TLC). Schizophr Bull (1986) 12(3):473–82 10.1093/schbul/12.3.473 3764363

[23] Green MF What are the functional consequences of neurocognitive deficits in schizophrenia? Am J Psychiatry (1996) 153(3):321–30 8610818 10.1176/ajp.153.3.321

[24] Keri S Szekeres G Kelemen O Antal A Szendi I Kovacs Z Abstraction is impaired at the perceptual level in schizophrenic patients. Neurosci Lett (1998) 243(1–3):93–6 10.1016/S0304-3940(98)00093-7 9535121

[25] Keri S Szekeres G Szendi I Antal A Kovacs Z Janka Z Category learning and perceptual categorization in schizophrenia. Schizophr Bull (1999) 5(3):593–600 10.1093/oxfordjournals.schbul.a033403 10478791

[26] Keri S Kelemen O Benedek G Janka Z Intact prototype learning in schizophrenia. Schizophr Res (2001) 52:261–4 10.1016/S0920-9964(00)00092-X 11705719

[27] Keri S The cognitive neuroscience of category learning. Brain Res Rev (2003) 43:85–109 10.1016/S0165-0173(03)00204-2 14499464

[28] Knowlton BJ Squire LR The learning of categories: parallel brain systems for item memory and category knowledge. Science (1993) 262(5140):1747–9 10.1126/science.8259522 8259522

[29] Squire LR Knowlton BJ Learning about categories in the absence of memory. Proc Natl Acad Sci U S A (1995) 92(26):12470–4 10.1073/pnas.92.26.12470 8618923 PMC40379

[30] Wechsler D Wechsler Adult Intelligence Scale-Revised Manual. San Antonio, TX: Psychological Corporation (1981).

[31] Missar CD Gold JM Goldberg TE WAIS-R short forms in chronic schizophrenia. Schizophr Res (1994) 12:247–50 10.1016/0920-9964(94)90034-5 8054316

[32] Kaufman AS Assessing Adolescent and Adult Intelligence. Needham, MA: Allyn & Bacon (1990).

[33] Jastak S Wilkinson GS The Wide Range Achievement Test-Revised Administration Manual. Wilmington, DE: Jastak Associates (1984).

[34] Kremen WS Seidman LJ Faraone SV Pepple JR Lyons MJ Tsuang MT The “3 Rs” and neuropsychological function in schizophrenia: a test of the matching fallacy in biological relatives. Psychiatry Res (1995) 56:135–43 10.1016/0165-1781(94)02652-1 7667438

[35] Dalby JT Williams R Preserved reading and spelling ability in psychotic disorders. Psychol Med (1986) 16:171–5 10.1017/S0033291700002609 3961042

[36] Frith CD Leary J Cahill C Johnstone E Performance on psychological tests: demographic and clinical correlates of the results of these tests. Br J Psychiatry Suppl (1991) 159(13):26–9 1840766

[37] Nelson HE McKenna P The use of current reading ability in the assessment of dementia. Br J Soc Clin Psychol (1975) 14:259–67 10.1111/j.2044-8260.1975.tb00178.x 1182406

[38] Nelson HE O’Connell A Dementia: the estimation of premorbid intelligence levels using the new adult reading test. Cortex (1978) 14:234–44 10.1016/S0010-9452(78)80049-5 679704

[39] Benton AL Hamsher KD Varney NR Spreen O Contributions to Neuropsychological Assessment. New York, NY: Oxford University Press (1983).

[40] Heaton RK Chelune GJ Talley JL Kay GG Curtiss G Wisconsin Card Sorting Test Manual. Odessa, FL: Psychological Assessment Resources (1993).

[41] Callicott JH Mattay VS Bertolino A Finn K Coppola R Frank JA Physiological characteristics of capacity constraints in working memory as revealed by functional MRI. Cereb Cortex (1999) 9:20–6 10.1093/cercor/9.1.20 10022492

[42] Delis DC Kramer JH Kaplan E Ober BA California Verbal Learning Test Manual. New York, NY: Psychological Corporation (1987).

[43] Gordon M McClure FD Aylward GP The Gordon Diagnostic System Interpretive Guide. 3rd ed DeWitt, NY: GSI Publications (1996).

[44] Reitan RM Trail Making Test: Manual for Administration and Scoring. Tucson, AZ: Reitan Neuropsychology Laboratory (1986).

[45] Wechsler D Wechsler Memory Scale-Revised Manual. New York, NY: Psychological Corporation (1987).

[46] Spreen O Strauss E A Compendium of Neuropsychological Tests. New York, NY: Oxford University Press (1991).

[47] Kay SR Fiszbein A Opler LA The Positive and Negative Syndrome (PANSS) for schizophrenia. Schizophr Bull (1987) 13:261–76 10.1093/schbul/13.2.261 3616518

[48] Elvevåg B Weickert T Wechsler M Coppola R Weinberger DR Goldberg TE An investigation of the integrity of semantic boundaries in schizophrenia. Schizophr Res (2002) 53(3):187–98 10.1016/S0920-9964(01)00202-X 11738532

[49] Smith EE Jonides J Working memory: a view from neuroimaging. Cogn Psychol (1997) 33(1):5–42 10.1006/cogp.1997.0658 9212720

[50] Fiez JA Raife EA Balota DA Schwarz JP Raichle ME Petersen SE A positron emission tomography study of the short-term maintenance of verbal information. J Neurosci (1996) 16(2):808–22 8551361 10.1523/JNEUROSCI.16-02-00808.1996 PMC6578642

[51] Manoach DS White NS Lindgren KA Heckers S Coleman MJ Dubal S Hemispheric specialization of the lateral prefrontal cortex for strategic processing during spatial and shape working memory. Neuroimage (2004) 21(3):894–903 10.1016/j.neuroimage.2003.10.025 15006656

[52] Walter H Bretschneider V Gron G Zurowski B Wunderlich AP Tomczak R Evidence for quantitative domain dominance for verbal and spatial working memory in frontal and parietal cortex. Cortex (2003) 39(4–5):897–911 10.1016/S0010-9452(08)70869-4 14584558

[53] Quan M Lee S-H Kubicki M Kikinis Z Rathi Y Seidman LJ White matter tract abnormalities between rostral middle frontal gyrus, inferior frontal gyrus and stratum in first episode schizophrenia. Schizophr Res (2013) 145:1–10 10.1016/j.schres.2012.11.028 23415471 PMC4110910

[54] Corkin S Beware of frontal lobe deficits in hippocampal clothing. Trends Cogn Sci (2001) 5(8):321–3 10.1016/S1364-6613(00)01709-5 11476991

[55] Kohler S Black SE Sinden M Szekely C Kidron D Parker JL Memory impairments associated with hippocampal versus para-hippocampal-gyrus atrophy: an MRI volumetric study in Alzheimer’s disease. Neuropsychologia (1998) 36(9):901–14 10.1016/S0028-3932(98)00017-7 9740363

[56] Keefe RS Silva SG Perkins DO Lieberman JA The effects of atypical antipsychotic drugs on neurocognitive impairment in schizophrenia: a review and meta-analysis. Schizophr Bull (1999) 25:201–22 10.1093/oxfordjournals.schbul.a033374 10416727

[57] Mishara AL Goldberg TE A meta-analysis and critical review of the effects of conventional neuroleptic treatment on cognition in schizophrenia: opening a closed book. Biol Psychiatry (2004) 55(10):1013–22 10.1016/j.biopsych.2004.01.027 15121486

[58] Weickert TW Goldberg TE First- and second-generation antipsychotic medication and cognitive processing in schizophrenia. Curr Psychiatry Rep (2005) 7(4):304–10 10.1007/s11920-005-0085-5 16098285

[59] Bonner-Jackson A Haut K Csernansky JG Barch DM The influence of encoding strategy on episodic memory and cortical activity in schizophrenia. Biol Psychiatry (2005) 58(1):47–55 10.1016/j.biopsych.2005.05.011 15992522 PMC1361687

[60] Lee TM Chan MW Chan CC Gao J Wang K Chen EY Prose memory deficits associated with schizophrenia. Schizophr Res (2006) 81(2–3):199–209 10.1016/j.schres.2005.08.009 16199138

[61] Ragland JD Gur RC Valdez JN Loughead J Elliott M Kohler C Levels-of-processing effect on frontotemporal function in schizophrenia during word encoding and recognition. Am J Psychiatry (2005) 162(10):1840–8 10.1176/appi.ajp.162.10.1840 16199830 PMC4332803

[62] Ragland JD McCarthy E Bilker WB Brensinger CM Valdez J Kohler C Levels-of-processing effect on internal source monitoring in schizophrenia. Psychol Med (2006) 36(5):641–8 10.1017/S0033291706007094 16608558 PMC4332577

[63] Schwartz BL Marvel CL Drapalski A Rosse RB Deutsch SI Configural processing in face recognition in schizophrenia. Cognit Neuropsychiatry (2002) 7(1):15–39 10.1080/13546800143000113 16528403 PMC1395477

[64] Calkins ME Gur RC Ragland JD Gur RE Face recognition memory deficits and visual object memory performance in patients with schizophrenia and their relatives. Am J Psychiatry (2005) 162(10):1963–6 10.1176/appi.ajp.162.10.1963 16199846

[65] Paul BM Elvevag B Bokat CE Weinberger DR Goldberg TE Levels of processing effects on recognition memory in patients with schizophrenia. Schizophr Res (2005) 74:101–10 10.1016/j.schres.2004.05.019 15694759

[66] Tracy JI Mattson R King C Bundick T Celenza MA Glosser G A comparison of memory for verbal and non-verbal material in schizophrenia. Schizophr Res (2001) 50:199–211 10.1016/S0920-9964(01)00241-9 11439241

[67] Brebion G David AS Bressan RA Pilowsky LS Word frequency effects on free recall and recognition in patients with schizophrenia. J Psychiatr Res (2005) 39(2):215–22 10.1016/j.jpsychires.2004.05.010 15589571

[68] Brebion G David AS Jones H Pilowsky LS Hallucinations, negative symptoms, and response bias in a verbal recognition task in schizophrenia. Neuropsychology (2005) 19:612–7 10.1037/0894-4105.19.5.612 16187879

[69] Egeland J Landro NI Sundet K Asbjornsen A Lund A Roness A Validation of distinct amnesic and executive type memory deficit in a psychiatric sample based on retrieval performance. Scand J Psychol (2005) 46(2):201–8 10.1111/j.1467-9450.2005.00449.x 15762947

[70] Kern RS Green MF Marshall BD Jr Wirshing WC Wirshing D McGurk SR Risperidone versus haloperidol on secondary memory: can newer medications aid learning? Schizophr Bull (1999) 25(2):223–32 10.1093/oxfordjournals.schbul.a033375 10416728

[71] Kim J Doop ML Blake R Park S Impaired visual recognition of biological motion in schizophrenia. Schizophr Res (2005) 77(2–3):299–307 10.1016/j.schres.2005.04.006 15922565

